# Feedback contributions to excitation–contraction coupling in native functioning striated muscle

**DOI:** 10.1098/rstb.2022.0162

**Published:** 2023-06-19

**Authors:** Samantha C. Salvage, Angela F. Dulhunty, Kamalan Jeevaratnam, Antony P. Jackson, Christopher L.-H. Huang

**Affiliations:** ^1^ Department of Biochemistry, University of Cambridge, Tennis Court Road, Cambridge CB2 1QW, UK; ^2^ Eccles Institute of Neuroscience, John Curtin School of Medical Research, The Australian National University, 131 Garran Road, Acton 2601, Australia; ^3^ Faculty of Health and Medical Sciences, University of Surrey, Daphne Jackson Road, Guildford GU2 7XH, UK; ^4^ Physiological Laboratory, University of Cambridge, Downing Street, Cambridge CB2 3EG, UK

**Keywords:** T-SR junction, ryanodine receptor, Na^+^ channel, C-terminal domains, III-IV linker, Ca^2+^ regulation

## Abstract

Skeletal and cardiac muscle excitation–contraction coupling commences with Na_v_1.4/Na_v_1.5-mediated, surface and transverse (T-) tubular, action potential generation. This initiates *feedforward*, allosteric or Ca^2+^-mediated, T-sarcoplasmic reticular (SR) junctional, voltage sensor-Cav1.1/Cav1.2 and ryanodine receptor-RyR1/RyR2 interaction. We review recent structural, physiological and translational studies on possible *feedback* actions of the resulting SR Ca^2+^ release on Na_v_1.4/Na_v_1.5 function in native muscle. Finite-element modelling predicted potentially regulatory T-SR junctional [Ca^2+^]_TSR_ domains. Na_v_1.4/Na_v_1.5, III-IV linker and C-terminal domain structures included Ca^2+^ and/or calmodulin-binding sites whose mutations corresponded to specific clinical conditions. Loose-patch-clamped native murine skeletal muscle fibres and cardiomyocytes showed reduced Na^+^ currents (*I*_Na_) following SR Ca^2+^ release induced by the Epac and direct RyR1/RyR2 activators, 8-(4-chlorophenylthio)adenosine-3′,5′-cyclic monophosphate and caffeine, abrogated by the RyR inhibitor dantrolene. Conversely, dantrolene and the Ca^2+^-ATPase inhibitor cyclopiazonic acid increased *I*_Na_. Experimental, catecholaminergic polymorphic ventricular tachycardic *RyR2-P2328S* and metabolically deficient *Pgc1β*^−/−^ cardiomyocytes also showed reduced *I*_Na_ accompanying [Ca^2+^]_i_ abnormalities rescued by dantrolene- and flecainide-mediated RyR block. Finally, hydroxychloroquine challenge implicated action potential (AP) prolongation in slowing AP conduction through modifying Ca^2+^ transients. The corresponding tissue/organ preparations each showed pro-arrhythmic, slowed AP upstrokes and conduction velocities. We finally extend discussion of possible Ca^2+^-mediated effects to further, Ca^2+^, K^+^ and Cl^−^, channel types.

This article is part of the theme issue ‘The heartbeat: its molecular basis and physiological mechanisms’.

## 1. Feedforward versus feedback events in striated muscle excitation–contraction coupling

Skeletal and cardiac muscle excitation–contraction coupling commences with Na^+^ channel, Na_v_1.4 or Na_v_1.5, mediated action potential (AP) initiation and propagation through their surface and transverse (T-) tubular membranes. Tubular Cav1.1 or Cav1.2 channels then sense the resulting voltage changes and transduce these into a ryanodine receptor (RyR1 or RyR2)-mediated sarcoplasmic reticular (SR) Ca^2+^ store release. This takes place through either reciprocal allosteric Cav1.1-RyR1 [1] or Ca^2+^-induced Cav1.2-RyR2 coupling in skeletal or cardiac muscle, respectively [2]. Release of intracellularly stored SR Ca^2+^ then results in the elevation of bulk cytosolic Ca^2+^ concentration, [Ca^2+^]_i_, that activates troponin, in turn triggering the myofilament action that causes myocyte contraction. However, in addition to these *feedforward* processes, recent speculations have considered possible *feedback* modulation of this process for which one example might involve a long loop Ca^2+^-mediated feedback signalling influencing the Na^+^ channel itself. This action would be consistent with the function of intracellular Ca^2+^ in acting as a strategic second messenger known to regulate widespread protein activity through numerous cell types.

We here review recent evidence for such a mechanism as applied to intact native functioning skeletal and cardiac muscle. We explore the possible anatomical and molecular background suggested by theoretical and experimental structural studies for such schemes, then examine evidence for its possible electrophysiological operation in native intact skeletal and cardiac muscle. These insights are translated into experimental and clinical human disease models. This involves first summarizing cell modelling and molecular background evidence bearing on the necessary diffusional and structural conditions of elevated Ca^2+^ and Na_v_1.4/Na_v_1.5 structures required for Ca^2+^ feedback action. These features are next related to results of loose-patch clamp studies exploring for alterations in voltage-dependent Na^+^ current densities, *I*_Na_, half-maximal activation and inactivation voltages *V*_1/2_, and steepness factors, *k*, following manipulations of intracellular [Ca^2+^] in native skeletal muscle fibres and cardiomyocytes. The latter employed the Exchange protein directly activated by cAMP (Epac) and RyR1/RyR2 activators, 8-(4-chlorophenylthio)adenosine-3′,5′-cyclic monophosphate (8-CPT) and caffeine, using as controls the RyR1/RyR2 inhibitor dantrolene, and the RyR1/RyR2 and Ca^2+^-ATPase inhibitors dantrolene and cyclopiazonic acid (CPA). Genetic disease and other chronic clinical models similarly permitted correlations between reduced *I*_Na_ and [Ca^2+^]_i_ changes and their specific pharmacological rescue. These included studies on murine gain of function, *RyR2-P2328S*, catecholaminergic polymorphic ventricular tachycardic (CPVT) and loss-of-function *Pgc1β*^−/−^ paradigms for pro-arrhythmic metabolic cardiac disease. Findings were related to physiological effects bearing on the consequent velocity of propagation of cell excitability and its translational consequences in intact organs. We finally discuss reports describing hydroxychloroquine (HCQ) challenge and the possibility of a Ca^2+^ modulation arising from altered Ca^2+^ release transients consequent upon prolonged AP recovery. We conclude speculating on possible roles for Ca^2+^-mediated feedback regulation as a general phenomenon that might similarly affect other, Ca^2+^, K^+^ and Cl^−^, channel activity *in vivo*.

## 2. Potential regulatory Ca^2+^ microdomains in the transverse tubule-sarcoplasmic reticular junction

**Figure 1.**
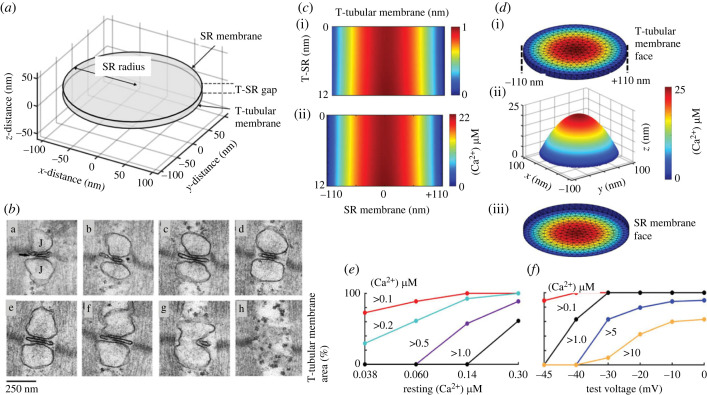
Model Ca^2+^ microdomain formation in the T-SR junction. (*a*) Formalized tubular (T) and SR membrane discs, radial (*xy*) diameter, *d* = 220 nm, separated by axial (*z*) T-SR distance, *w* = 12 nm, representing (*b*) amphibian muscle T-SR junction geometry, shown in serial electron microscope sections (a–h). (*c*,*d*) [Ca^2+^] modelling results in (*c*) midline axial views in (i) resting and (ii) activated amphibian skeletal muscle, and (*d*) (i) radial and (ii) perspective T-tubular and (iii) radial SR membrane mapping of [Ca^2+^] following activation. (*e,f*) Proportions of tubular membrane exposed to different [Ca^2+^]_i_ (*e*) in resting muscle assuming different resting bulk cytosolic [Ca^2+^]_i_ and (*f*) following activation to different membrane potentials *V*_m_ (from figure 2 of Martin *et al.* [6] and figures 1 and 2A,B,D,F, 7C and 8a,B of Bardsley *et al.* [7] by permission).

Structural descriptions of skeletal or cardiac muscle, sarcomeres and their surface, T-tubular and SR membranes localize the processes coupling T-tubular membrane potential changes and SR Ca^2+^ release to T-SR triadic or dyadic junctions [3–5]. These constitute regions where Na_v_1.4/Na_v_1.5-expressing T-tubular membranes come into close geometrical relationship with, while remaining electrically isolated from, the RyR1/RyR2-expressing terminal cisternal SR membranes. They thus form regions where both feedforward and Ca^2+^-mediated feedback actions could take place. Their quantitative detailed anatomy appears compatible with the latter function. It can be quantified (figure 1*a*) using findings from serially sectioned electron microscope images, here exemplified for amphibian skeletal muscle fibres (figure 1*b*) [6]. T-SR junctional regions taking the form of triad or dyad junctions in skeletal or cardiac muscle, respectively, form restricted diffusion spaces bounded by their component T-tubular or SR membranes in parallel, approximately 100–400 nm, alignment. These would potentially permit ion accumulation or depletion. Here, local Ca^2+^ release might elevate their [Ca^2+^]_TSR_ to levels sufficient to mediate regulatory feedback effects.

Fluorescent Ca^2+^ indicator results were compatible with such [Ca^2+^]_TSR_ microdomains with their small dimensions and dispersed nature [8–10]. These compared experimental kinetic and steady state signals from introduced Ca^2+^ sensors localized to T-SR gap regions. This involved Ca^2+^ sensor conjugation to FKBP12.6 [8], triadin [10] or the Ca_v_1.1 *γ*1 auxiliary subunit [10]. This permitted comparisons of their [Ca^2+^] with those existing in the bulk cardiac [8] or skeletal muscle cytosol [10], albeit admitting unproven assumptions of equal cleft and bulk concentrations of the Ca^2+^ probes [10]. The findings suggested differing resting T-SR regional (194 nM) and bulk cytosolic [Ca^2+^] (100 nM) attributable to background SR Ca^2+^ leak [8]. Following activation of excitation–contraction coupling, fluorescence from untargeted Ca^2+^ probes was distributed throughout the muscle fibres with large Ca^2+^-dependent changes and obvious kinetic delays [10]. T-SR junctional [Ca^2+^] more rapidly reached distinct maximum values distinctly higher [8] or lower [10] compared to corresponding findings in bulk [Ca^2+^]. These findings prompted preliminary modelling explorations of the functions of possible dedicated and promiscuous calmodulin pools, respectively, directly regulating channel activity and sensing local Ca^2+^ to trigger downstream Ca^2+^/CaM-dependent protein kinase II and calcineurin [9].

Subsequent quantitative, finite-element diffusional modelling of RyR1/RyR2-mediated Ca^2+^ release and its subsequent fluxes and consequent concentrations through the T-SR junction used as geometrical parameters their established distributions, densities and ultrastructure in amphibian skeletal muscle [7]. Such modelling employed previously experimentally quantified SR Ca^2+^ release rates in response to varying test voltage steps [11]. It predicted that T-SR junctional regions could both accumulate and deplete released SR Ca^2+^ in both activated and resting muscle fibres [7]. The predicted RyR-mediated Ca^2+^ influx densities *J*_influx_ across the SR membrane face of each T-SR junction gave rise to a Ca^2+^ diffusion through a radially symmetric T-SR junctional space (figure 1*c,d*). This was followed by its first-order diffusional efflux at the edge of the modelled junction into the well-stirred cytosolic space. Here the modelling correctly replicated previously observed bulk cytosolic [Ca^2+^]_i_, before similarly replicating their eventual first-order SERCA-mediated Ca^2+^ SR re-sequestration [12].

The resulting Ca^2+^ microdomains showed local [Ca^2+^]_TSR_ ranging from approximately 0.3–0.4 to 17–20 µM at voltages corresponding to contraction threshold and full activation, respectively. These traversed the entire axial T-SR distance (figure 1*c*) with radial, greater than fivefold, [Ca^2+^] differences between T-SR junction centre and edge (figure 1*d*i–iii). The Ca^2+^ microdomains robustly persisted through 10^4^-fold variations in Ca^2+^ diffusion coefficient, and fivefold variations in T-SR distance and diameter, at constant *J*_influx_, and 10- and 100-fold *J*_influx_ reductions below threshold levels of Ca^2+^ release. Finally, they also persisted giving T-SR centre [Ca^2+^] at μM levels at *J*_influx_ corresponding to reported bulk cytosolic [Ca^2+^]_i_ even in resting as opposed to activated muscle [13–15]. Significant proportions of resting T-tubular membrane then experienced significant, approximately 0.5 µM, [Ca^2+^] through typical reported resting bulk cytosolic [Ca^2+^] values (figure 1*e*). In such simulations, muscle activation by successively larger depolarizing steps recruited correspondingly greater proportions of T-tubular membrane that experienced successively higher, 0.1 to 10 µM, [Ca^2+^] (figure 1*f*).

The resulting local [Ca^2+^]_TSR_ microdomains generated by RyR-mediated Ca^2+^ release could then access T-tubular Na_v_1.4 or Na_v_1.5 channels in the T-SR junction vicinity. The available evidence could prompt similar future studies extending to cardiac Na_v_1.5 as opposed to skeletal muscle Na_v_1.4 channels. Mammalian atrial and ventricular cardiomyocyte junctions between surface and SR membranes differ from those in skeletal muscle in involving peripheral couplings or dyadic rather than triadic junctions [4]. Nevertheless the detailed organization and separation of their participating T-tubular and SR membranes remain similar and therefore are similarly compatible with junctional Ca^2+^ domain formation [16].

## 3. Ca^2+^ and Ca^2+^-calmodulin regulatory domains in voltage-sensitive Na^+^ channels

**Figure 2.**
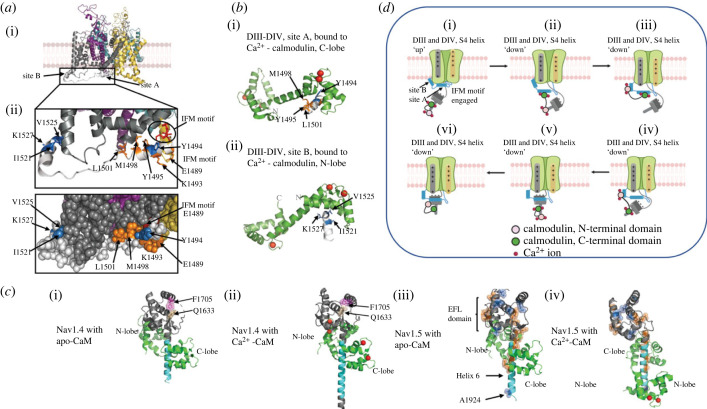
Regulatory-binding sites on the voltage-gated Na^+^ channels. (*a*) Na_v_1.5 channel showing DIII-DIV linker region in grey (i) and at closer resolution (ii) with binding sites A and B marked. (*b*) Ca^2+^-calmodulin (CaM) C-lobe (i) and N-lobe binding (ii), respectively, to site A and site B helices. Residues associated with clinical phenotypes marked and coloured. (*c*) Apo-CaM (i,iii) or Ca^2+^-CaM (ii, iv) binding to Na_v_1.4 (i, ii) and Na_v_1.5 (iii, iv) CTDs in side view. Ca^2+^ shown as red balls. Myotonia and PMC-associated residues highlighted in tan and purple in the Na_v_1.4 structures (i,ii). LQTS and BrS-associated residues highlighted in orange and sky blue, respectively, in the Na_v_1.5 structures (iii,iv). (*c*) CaM-binding patterns and possible actions on Na_v_1.5 recovery from inactivation. (*d*) (i) IFM motif and DIII-DIV linker fully engaged with *α* subunit; CTD dissociated from site A in the inactivated state with Ca^2+^-CaM bound to helix 6 (PDB structure 4jq0). (*d*) (ii)–(v) Conformational interactions arranged in a possible sequential channel recovery from inactivation. (vi) Proposed Na_v_1.5 conformation after return to resting state. If Ca^2+^ levels are low, the predominant apo-CaM binds to helix 6 (PDB structure 4ovn) (By permission from figures 2b,C, 3B, 4B of Salvage *et al.* [17]).

Recent cryo-electron microscope characterizations of Na_v_1.4/Na_v_1.5 structure are consistent with an existence of molecular regions that might be involved in a Ca^2+^-mediated feedback modulation. These are distinct from regions previously implicated in rapid channel activation events. However, they are compatible with interactions with those Na_v_1.4/Na_v_1.5 regions involved in the subsequent, slower, channel inactivation events. Excitation–contraction coupling *feedforward* mechanisms thus commence with Na_v_1.4 or Na_v_1.5 activation by voltage-sensitive outward transitions of positively charged residues in voltage-sensing S4 helices in the outer rim of their domains DI-III (figure 2*a*i; [18–21]). Coupled conformational changes in the channel pore, S5 and S6 helices, then drive the resting, *closed* to *open*, activated transition, permitting the inward, depolarizing, transmembrane Na^+^ fluxes initiating cell excitation. However, activation is then followed by channel pore closure into an *inactivated* state [18,22]. This is the consequence of slower outward DIV S4 helix movements. These allow binding of a hydrophobic IFM (isoleucine, phenylalanine and methionine) motif within the cytoplasmic III-IV linker to a hydrophobic pocket between domains III and IV (figure 2*a*ii). The latter entities could be involved in additional *feedback* Ca^2+^ regulatory events through further interactions with a globular, intracellular C-terminal domain (CTD) flexibly connected to the Na_v_ DIV S6 helix.

CTDs begin at residues 1599 on Na_v_1.4 and 1773 on Na_v_1.5 with a sequence of five α-helical regions fitting the EF-like hand (EFL) consensus sequence [23], a sixth α-helical region and further more disordered and less-well structurally characterized regions. The EFL contains a prominent cleft bounded by helices 1 and 5, with helix 4 forming its floor [24]. In resting Na_v_1.x, this can form complexing salt bridges with site A (residues 1490–1503) of the DIII-IV linker constraining the IFM motif [25,26]. With channel opening and outward movement of the DIV S4 helix, the salt bridges are disrupted. This dissociates CTD from the DIII-DIV linker, freeing the IFM motif to transition into the inactivated state. The latter allows restoration of the resting membrane potential (figure 2*a*ii).

Ca^2+^-binding acidic residues of Na_v_1.5 CTD EFLs [23,27,28] lie along the outward face of helix 1 rather than within the turn loops between adjacent α-helices elsewhere associated with high Ca^2+^ affinity [24,29,30]. Nevertheless, both DIII-IV linker and CTD show potential regulatory sites that can bind the established nM-low µM K_d_ Ca^2+^ sensor calmodulin, CaM [31–33]. CaM includes N- and C-lobes each possessing two Ca^2+^-binding EF hands (figure 2*b*i,ii). Ca^2+^-free, apo- and Ca^2+^ bound, and Ca^2+^-CaM, respectively, show ‘closed’ and ‘semi-open’ and ‘open’ and ‘semi-open’ states. The ‘open’ and ‘semi-open’ states show EF hand helix orientations exposing a hydrophobic groove able to bind particular α-helical protein sequence motifs.

The various binding properties of these N- and C- lobes of apo- and Ca^2+^-CaM respectively to the DIII-DIV linker and CTD give rise to a range of possible interactions between CaM and the Na_v_1.4/Na_v_1.5. First, Ca^2+^-CaM but not apo-CaM, via its N- and C-lobes, binds to isolated, purified *DIII-DIV linker domains* at sites B (1500–1530) and A (1490–1503), respectively (figure 2*b*i,ii) [34,35]. The latter is additional to site A's ability to bind the CTD EFL cleft. Na_v_1.5 DIII-DIV linker and CTD co-precipitation is thus catalysed by Ca^2+^-CaM, but inhibited by the Ca^2+^-chelator, EGTA [36]. Second, CTDs bind the *CaM* with potential regulatory effects [37,38]. The IQ motif within helix 6 of the Na_v_1.4 or Na_v_1.5 [39] CTD EFL [40] can bind the ‘semi-open state’ apo-CaM C-lobe [31]. Additionally, both helix 3 and the N-terminal of helix 6 can bind the ‘closed-state’ apo-CaM N-lobe (figure 2*c*i,iii). The IQ motif (figure 2*c*ii,iv) can also bind the ‘semi-open state’ Ca^2+^-CaM C-lobe. In Na_v_1.5, a downstream slightly overlapping N-lobe-binding motif (NLBM) can then bind a shifted Ca^2+^-CaM N-lobe (figure 2*c*iv). Na_v_1.4 contrastingly lacks a functioning NLBM [41] leaving the Ca^2+^-CaM N-lobe free to interact elsewhere, potentially the III-IV linker B-site (figure 2*c*ii) [35,42,43]. In either event, simultaneous binding of either site A or B of the DIII-DIV linker, to their respective Ca^2+^-CaM C or N-lobes, and the remaining α-subunit in an inactivated Na_v_1.4 or Na_v_1.5 thus becomes unlikely.

These binding properties result in a variety of potential interaction patterns between apo- and Ca^2+^ calmodulin, and the DIII-DIV linker and CTD of Na_v_1.4/Na_v_1.5. The Ca^2+^-CaM then could bind to the CTD in an inactivated Na_v_1.5 (figure 2*d*i). Freeing sites A and B to permit such binding would require IFM motif disengagement from its binding site (figure 2*d*ii). The binding could involve the pair of Ca^2+^-CaM N and/or C lobes, respectively, capable of binding exclusively to either the III-IV linker (figure 2*d*iii; [35]) or the CTD (figure 2*d*i,v,vi; [30]). Alternatively the Ca^2+^-CaM could cross-link between them whether involving the Na_v_1.5 NLBM (figure 2*d*ii; [34]) or Na_v_1.4 IQ domain (figure 2*d*iv; [44]).

These patterns could form a speculative recovery sequence from inactivation to the channel resting state involving modulation through direct or CaM-mediated Ca^2+^ binding (figure 2*d*i–vi) [17]. A binding of one or both Ca^2+^-CaM lobes to the DIII-DIV linker (figure 2*d*iii; [35]) might then potentiate recovery from inactivation. Conversely, Ca^2+^ binding to the CaM C-lobe reduces its affinity for the CTD [34,42] enhancing Na_v_1.5 inactivation (figure 2*d*ii) [34]. Finally, Ca^2+^-CaM binding to the III-IV linker could also modify recovery from inactivation by precluding (figure 2*d*ii–iv) direct CTD association with the III-IV linker (figure 2*d*v,vi).

An existence of further potentially regulatory Na_v_1.5 channel sites may be reflected in increases in late Na^+^ current (*I*_NaL_) causing delayed AP repolarization as occurs in long QT type 3 syndrome (LQTS3). Effects of KN93 on these implicate its action on CaMKII [45] or CaM on phosphorylation of particular (Ser516, Ser571 and Thr594) DI-DII intracellular linker residues, or on CaM-III-IV linker interaction [35]. Phosphorylation at a protein kinase C-specific site reduced peak *I*_Na_ and shifted steady state inactivation *V*_1/2_ (by −15 mV) [46]. Finally, Na_v_1.5 N-terminal domain CaM-binding site mutations have been attributed to a downregulation of *I*_Na_ [47].

## 4. Na^+^ channel inhibition by RyR-mediated Ca^2+^ release

Following RyR-mediated SR Ca^2+^ release induced by AP initiation in native skeletal muscle fibres and cardiomyocytes, bulk cytosolic [Ca^2+^]_i_ increases from approximately 100 nM to 1–10 µM [13–15]. The latter concentrations affected Na^+^ channel function in studies using *in vitro* expression systems [48]. Here, N-(2-hydroxyethyl)ethylenediamine-N,N′,N′-triacetic acid mediated Ca^2+^ buffering (*K*_d_ = 4 µM) permitting [Ca^2+^]_i_ approximately 10 µM to be reached; Nitr-photo-Ca^2+^ uncaging and activating co-expressed Cav1.2 gave concordant results. All reduced maximum *I*_Na_ in HEK293 cells expressing Na_v_1.4, or Na_v_1.5 chimeras with the Na_v_1.4 CTD, but not those expressing Na_v_1.5 or Na_v_1.4 chimeras with the Na_v_1.5 CTD [42,48]. However, freshly isolated rabbit [49] and cultured rat ventricular myocytes [50] showed reduced *I*_Na_ with [Ca^2+^]_i_ elevation produced by patch electrode Ca^2+^-BAPTA or caffeine challenge and 24 h sustained culture medium [Ca^2+^] elevations, respectively. Inactivation *V*_1/2_s was unaffected through all these studies [48]. These differences underline the importance of conducting experimental analyses in intact native systems.

Furthermore, the modelling studies above suggested that *in vivo* [Ca^2+^]_TSR_ could reach still higher levels, sufficient for regulatory Ca^2+^-CaM binding. Intact functioning native skeletal muscle fibres and cardiomyocytes both also demonstrated corresponding *I*_Na_ alterations during loose-patch clamp recording under conditions of perturbed Ca^2+^ homeostasis [51]. The latter technique avoided membrane disruptions and perturbations of [Ca^2+^]_i_ homeostasis arising from the measurement procedures themselves. Altered [Ca^2+^]_i_ homeostasis could accompany and affect results from other experimental studies, including conventional patch clamp, which further often employ Ca^2+^-chelating EGTA- and F^−^-containing pipette solutions. Furthermore, loose-patch clamping additionally permits sequential and multiple recordings to be made with the same standardized pipette before and after pharmacological challenge, and studies to be made in intact *in situ* skeletal muscle fibres and cardiomyocytes, as opposed to isolated or cultured cells. Features of their reported Na^+^ current, *I*_Na,_ concurred with those obtained on earlier occasions and with results from other voltage clamp methods applied to mammalian skeletal muscle fibres [51,52]. Voltage steps from resting to sequentially depolarized test potentials provided families of *I*_Na_ activation records. Superimposed further pulses to a fixed depolarized level could determine the voltage dependences of the consequent channel inactivation. Records were compared before and following the perturbations of *in vivo* Ca^2+^ homeostasis.

**Figure 3.**
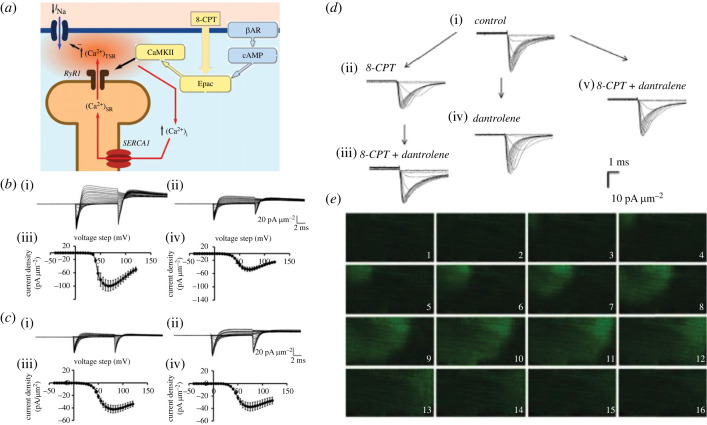
8-(4-chlorophenylthio)adenosine-3′,5′-cyclic monophosphate (8-CPT) effects on Na^+^ channel activation. (*a*) Scheme summarizing RyR activation through 8-CPT agonist action on Epac-mediated cAMP signalling. (*b–d*) Experimental loose-patch clamping results. (*b*,*c*) Ion currents in response to activating voltage steps in native murine skeletal muscle showing current families (i) before and (ii) following 8-CPT challenge. Results compared (*b*) before and (*c*) following introduction of dantrolene. (iii,iv) Corresponding current–voltage relationships. (*d*) Ion currents in experimental-isolated murine ventricular preparations (i) in the absence and (ii) presence of 8-CPT challenge before and (iii) following further addition of dantrolene, and (iv) in the presence of dantrolene alone and (v) combined with 8-CPT. (*e*) Isolated ventricular myocyte; fluo-3 images under confocal microscopy: Epac-induced wave of elevated cytosolic [Ca^2+^]. Successive 41.0 × 20.5 µm frames each separated by 65 ms. ((*b*) From figure 3a*–*d, (*c*) from figure 5a*–*d of Matthews *et al.* [58]; (*d*) from figure 2 of Valli *et al.* [59]; (*e*) from Figure 8 of Hothi *et al.* [54] by permission.)

Experiments manipulating RyR-mediated intracellular SR store Ca^2+^ release demonstrated potentially physiologically significant negative feedback regulations of Na_v_1.4 and Na_v_1.5 in both skeletal muscle fibres and cardiomyocytes. These employed challenge by the two RyR-mediated SR Ca^2+^ release agonists 8-CPT [53,54] and caffeine [55] and the SR Ca^2+^-ATPase inhibitor CPA [56]. In addition, the RyR antagonist dantrolene [57] was used both by itself and in combination with those remaining agents in control studies. The latter use of agents with antagonistic actions focused their actions on their effects specifically upon local cytosolic [Ca^2+^]. Exchange protein directly activated by cAMP (Epac) causes a downstream RyR phosphorylation stimulating SR Ca^2+^ release (figure 3*a*). In murine skeletal muscle fibres, the Epac activator 8-CPT (1 µM) [53,54] reduced maximum inward *I*_Na_ by 30–50% (figure 3*b*i,ii). RyR block by dantrolene (10 µM) abrogated these 8-CPT actions leaving *I*_Na_ unchanged (figure 3*c*i,ii) [58]. Both activation and inactivation half-maximal voltages *V*_1/2_ remained unchanged through such manoeuvres (figure 3*b*iii,iv;*c*iii,iv).

8-CPT challenge similarly reduced peak *I*_Na_ in murine cardiac atrial and ventricular myocyte preparations (figure 3*d*i,ii) [59]. By contrast, peak *I*_Na_ became indistinguishable from control levels with further addition of dantrolene (10 µM; figure 3*d*iii), or in the presence of dantrolene whether alone (figure 3*d*iv) or combined with 8-CPT (figure 3*d*v). Activation and inactivation *V*_1/2_ and *k* and time constants for *I*_Na_ recovery from inactivation remained constant through these manoeuvres [59]. 8-CPT-treated murine atrial myocytes correspondingly demonstrated spontaneous cytosolic Ca^2+^ ([Ca^2+^]_i_) transients, increased amplitudes of [Ca^2+^]_i_ transients evoked by AP excitation, and spectrofluometrically measurable spontaneous Ca^2+^ waves suggesting Ca^2+^ homeostatic changes (figure 3*e*) [54]. Finally, the same isolated hearts provided intact Langendorff-perfused preparations to assess the physiological consequences of these procedures. These studies demonstrated reduced maximum rates of atrial and ventricular AP upstroke, (d*V*/d*t*)_max_, increased AP latencies and ventricular arrhythmic phenotypes on rapid pacing or extrasystolic stimuli with intracellular sharp microelectrode membrane potential recordings [59,60].

## 5. Reciprocal Na_v_1.4 alterations following Ca^2+^ store release and depletion

**Figure 4.**
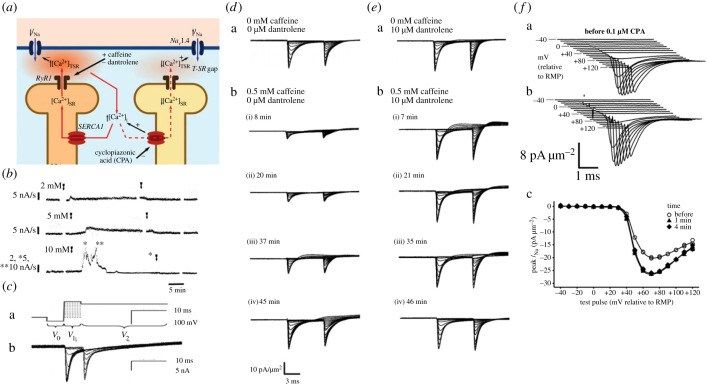
Paradoxical, reciprocal Na^+ ­^current increases and decreases following caffeine and CPA-induced Ca^2+^ store release and depletion in skeletal muscle. (*a*) Possible action of caffeine induced RyR-mediated release followed by the depletion of intracellularly stored Ca^2+^ (left), and CPA-mediated store Ca^2+^ depletion by SERCA inhibition (right). This explains (*b*) varying actions of 2–10 mM caffeine on rat fast twitch muscle background aequorin Ca^2+^ signals at 25°C (arrows delimit periods of caffeine exposure) and corresponding effects of (*c*) the actions of (a) the double pulse protocols imposing families of varying depolarizing activating steps each succeeded by a step to a constant 95 mV depolarization on (b) the resulting families of loose-patch clamped murine skeletal muscle membrane currents. (*d*,*e*) These are illustrated (a) before and (b) at successive intervals ((i)–(iv)) after adding caffeine (0.5 mM), (*d*) before and (*e*) following addition of dantrolene (10 µM) (current densities in pA µm^−2^). (*f*) Membrane currents (a) before and (b) 1 min following introduction of CPA (0.1 µM) with (c) consequent changes in I–V relationships ((*b*) from figure 2 of Fryer & Neering [61]; (*c*) from figure 1 of Valli *et al.* [59]; (*d,e*) from figure 3 of Satbjit-Singh *et al.* [62]; (*f*) from figure 3a,b,d of Liu *et al.* [63] by permission).

Independent pharmacological manipulations of intracellular skeletal muscle fibre Ca^2+^ homeostasis, potentially altering [Ca^2+^]_TSR_ (figure 4*a*), yielded concordant results [62]. The RyR agonist caffeine, applied at 0.5 and 2 mM, respectively, produces a sustained activation, and a transient activation followed by inactivation, of RyR-mediated SR Ca^2+^ release, correspondingly altering [Ca^2+^]_i_ in mammalian skeletal muscle fibres (figure 4*b*) [61]. Electrophysiological studies showed parallel, similarly time-dependent, decreases (figure 4*d*b) and increases in *I*_Na_ when 0.5 and 2 mM caffeine was added before but not following establishing the loose-patch clamp seal (figure 4*c*). Dantrolene (10 µM) abrogated these effects (figure 4*e*). Applied by itself it induced small *I*_Na_ increases suggesting that it relieved inhibitory effects of a persistent resting Ca^2+^ release (figure 4*d*a,*e*a) [62].

The SR Ca^2+^-ATPase inhibitor CPA (0.1 and 1 µM) has been similarly reported to increase bulk cytosolic [Ca^2+^]_i_ through a contrasting mechanism inhibiting SR Ca^2+^ re-uptake (figure 4*a*). However, *I*_Na_ now *increased* by approximately 30% (figure 4*f*a,b), with insignificant effects on *V*_1/2_ and *k* (figure 4*f*c), within 1–4 min of introducing CPA-containing but not control solution through a bespoke flow system in identical stable membrane patches in skeletal muscle fibres [63]. CPA pre-treatment also abrogated the previously reported reductions in *I*_Na_ produced by 0.5 mM caffeine. In complementing results of directly modifying SR Ca^2+^ release, these paradoxical findings are compatible with reduced RyR-mediated background fluxes of SR Ca^2+^ into a T-SR [Ca^2+^]_i_ microdomain. This could result from SR Ca^2+^ depletion and would transiently reduce local [Ca^2+^]_TSR._ These findings thus together implicate possible local T-SR domains in which [Ca^2+^]_TSR_ was either increased or reduced in such Na_v_1.4 down- or upregulation [63]. Finally, prolonged increases in extracellular [Ca^2+^], caffeine and CPA, expected to elevate [Ca^2+^]_i_, also reduced mean peak inward *I*_Na_ in murine atrial preparations [64].

## 6. Potential feedback roles in normal and abnormal skeletal muscle excitation–contraction coupling *in vivo*

The time course of the possible feedback action on Na_v_1.4/Na_v_1.5 by released Ca^2+^ is likely to significantly lag the electrical events within single individual AP activation and recovery cycles. Thus, within a single AP, the skeletal or cardiac muscle Na_v_1.4- or Na_v_1.5-mediated surface membrane excitation precedes activation of surface Ca^2+^ channel-mediated extracellular Ca^2+^ entry and/or RyR-mediated SR Ca^2+^ release. Such a timing would also extend to the significantly earlier AP recovery in skeletal muscle. The Na_v_1.4 or Na_v_1.5 likely subsequently undergoes inactivation before, or early within, the time course of the immediately ensuing Ca^2+^ transient, apart from possible prolonged late *I*_NaL_ current previously associated with Na_v_1.5 [65]. Finally, following recovery of the cytosolic Ca^2+^ transient, much of this bound Ca^2+^ would dissociate from either the CaM or the Na_v_ itself, before the following AP upstroke.

Nevertheless such Ca^2+^-mediated feedback could influence membrane excitability through the more prolonged time course of sustained, repetitive, AP activity. Thus, during cycles of repetitive AP firing, longer term, background, [Ca^2+^]_i_ could, respectively, increase or decrease at sustained high- or low-firing rates. This could modify Na_v_1.4/Na_v_1.5 function either directly or through Ca^2+^-CaM diminishing or enhancing Na_v_1.4/Na_v_1.5 availability for driving AP upstroke and propagation resulting in a form of Ca^2+^ memory. [Ca^2+^]_i_ could also increase with compromised SR Ca^2+^-ATPase activity following ATP depletion. In skeletal muscle, this could result in physiologically adaptive reductions in cell excitability permitting recovery from fatiguing stimulation [66]. Such effects of [Ca^2+^]_i_ could be physiologically important particularly under conditions of intense and prolonged muscle activation including tetanic stimulation and metabolic exhaustion. For example, tetanic stimulation in amphibian muscle was followed by significantly prolonged decays in [Ca^2+^]_i_ during recovery, extending even beyond full muscle relaxation. These processes could elevate [Ca^2+^]_i_ to even approximately 1 µM levels. Both these features were accentuated by increasing stimulation duration [67].

The presence of a Ca^2+^-mediated feedback would predict consequences for Na_v_1.4/Na_v_1.5 function with clinical mutations involving the relevant functional components of either the Na^+^ channel or the RyR1/RyR2. Sustained alterations in such properties appear in electrophysiological disease phenotypes associated with Na_v_1.4 and Na_v_1.5, CTD and III-IV linker mutations [68]. In skeletal muscle, a number of genetic Na_v_1.4 mutations accompanying K^+^ and cold-aggravated myotonias are associated with reduced Ca^2+^-dependent Na^+^ channel inhibition [48]. One such, K^+^-aggravated, myotonia accompanied Na_v_1.4 mutations in its EF-hand-like domain and was associated with slowed *I*_Na_ kinetics and impaired *I*_Na_ inactivation [69]. A further Na_v_1.4 CTD variant clinically associated with human cold-aggravated myotonia manifesting as a transient loss of fibre excitability, myotonic stiffness and a periodic paralysis contains two amino acid substitutions, DI S5-S6 loop T323M and CTD F1705I. Whole-cell patch clamp *I*_Na_ in HEK293 cells expressing Na_v_1.4-T323M were indistinguishable from wild-type (WT), consistent with a benign polymorphism. However, Na_v_1.4-F1705I was associated with normal *I*_Na_ activation but slowed fast inactivation with a +8.6 mV shifted *V*_1/2_ [70]. Similarly, one report on cultured human malignant hyperthermic skeletal myocytes with particular gain-of-function RyR1 mutations described increased slowly inactivating inward, tetrodotoxin-sensitive current [71]. Finally, the weakness observed in dystrophic muscle is associated with an elevated [Ca^2+^]_i_ [55].

## 7. Ca^2+^-dependent Na_v_1.5 modulation in cardiac disease models

In cardiac muscle, reductions in AP conduction velocity (CV) consequent upon loss of Na_v_1.5 function have been implicated in specific pathological pro-arrhythmic phenotypes. Thus, some cases of the pro-arrhythmic loss of Na_v_1.5 function Brugada Syndrome (BrS) show mutations in site B of the DIII-DIV linker [72] (figure 2*b*ii), or in the CTD, particularly its EFL sites. These could compromise capture of the DIII-DIV linker during recovery from inactivation, and of the NLBM (figure 2*a*). Contrastingly, some gain of Na_v_1.5 function LQTS3 cases show DIII-DIV linker, site A mutations affecting residues stabilizing DIII-DIV linker binding to the inactivation site (figure 2*b*i). Other LQTS3 cases show CTD, helix 6 mutations particularly affecting the IQ motif anchoring apo-CaM (figure 2*c*iii,iv) [72]. The latter are rescued by an overexpressed CaM [73]. Both could compromise channel inactivation [72]. The one site A (Y1496) BrS-related mutation points away from the inactivation site and strongly contacts the Ca^2+^-CaM C lobe [34].

**Figure 5.**
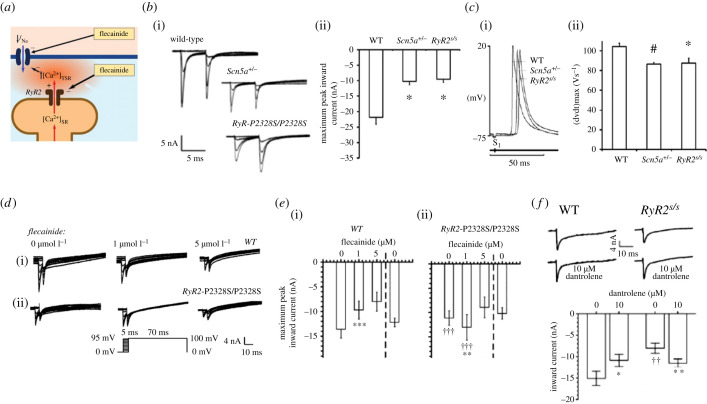
Na^+^ current reduction in murine gain-of-function RyR2-P2328S/P2328S CPVT model and its paradoxical flecainide rescue. (*a*) Scheme for action of increased SR store Ca^2+^ release, summarizing: (*b*) loose-patch clamp membrane current results showing (i) families of WT, *Scn5a*+/− and *RyR2*-P2328S/P2328S atrial currents obtained by double pulse protocols (figure 4*c*i), and (ii) their maximum peak inward currents (*p < 0.005); and (*c*) the resulting (i) left atrial intracellular AP waveforms showing their conduction delays reflecting (ii) their varying maximum upstroke rates. (*d*) Membrane currents in 0, 1 and 5 µM flecainide in (i) WT and (ii) *RyR2-P2328S/P2328S* left atria. (*e*) Their respective (i,ii) maximum peak currents with exposure followed by the withdrawal of flecainide. (*f*) Parallel effects in membrane currents in response to an 80 mV depolarizing step before and following dantrolene (10 µM) challenge ((*b*) from figure 5C and (*c*) from figure 5A of King *et al.* [64]; (*d*) from figure 4(a) and (b), (*e*) from figure 3(c) and (d), (*f*) from figure 3, inset of Salvage *et al.* [76] by permission).

Cardiac *RyR2*, calsequestrin (*CASQ2*), triadin (*TRDN*) or calmodulin (*CALM1*, *CALM2* and *CALM3*) mutations [74] are associated with CPVT. This predisposes to potentially fatal mono-, polymorphic or bidirectional ventricular tachycardic episodes following adrenergic stress, as well as to atrial fibrillation [75]. Experimental murine hetero- and homozygotic *RyR2-P2328S* ventricular cardiomyocytes showed abnormal RyR2-mediated diastolic [Ca^2+^]_i_ elevations (figure 5*a*) [77]. Bilayer homozygotic *RyR2-P2328S* channels correspondingly exhibited enhanced channel activity at a cytoplasmic [Ca^2+^] of 1 µM as well as greater than 10-fold negative and greater than 1000-fold positive shifts in cytosolic Ca^2+^ dependences of activation and inactivation, respectively, but normal unit channel conductances [78].

Altered *I*_Na_ potentially could contribute to the CPVT phenotype associated with the *RyR2-P2328S* variant. Loose-patch clamp measurements in superfused homozygotic *RyR2-P2328S* atria revealed markedly reduced peak *I*_Na_ to extents comparable to findings in Na_v_1.5-haploinsufficient *Scn5a*^+/−^ atria (figure 5*b*i,ii). Activation and inactivation current–voltage relationships were unchanged [64]. Floating intracellular microelectrode measurements correspondingly demonstrated reduced interatrial CVs and (d*V*/d*t*)_max_, but normal recovery, AP duration (APD) and refractory period values (figure 5*c*i,ii). Multi-electrode array recordings confirmed reduced atrial epicardial AP CVs [79]. Both spontaneously beating and regularly (S1) paced *RyR2-P2328S* but not WT atria correspondingly showed sustained tachyarrhythmias, delayed afterdepolarizations and ectopic APs. These likely resulted from the compromised CVs. Thus, extrasystolic, S2, stimulation provoked arrhythmia at longer S1S2 intervals in the *RyR2-P2328S* that nevertheless corresponded to similar (d*V*/d*t*)_max_, and effective interatrial CVs as those provoking arrhythmia in WT [79]. Spontaneously beating *RyR2-P2328S* hearts also recapitulated clinical pro-arrhythmic ventricular phenotypes on isoproterenol and caffeine challenge [80]. These correlated with reduced ventricular (d*V*/d*t*)_max_ and epicardial CVs, particularly in homo- as opposed to heterozygotic *RyR2-P2328S* and WT hearts [80].

## 8. A translational basis for flecainide-based catecholaminergic polymorphic ventricular tachycardic monotherapy

Rescue of the conduction slowing resulting from a Ca^2+^-mediated reduction in *I*_Na_ may offer a basis for recent introduction of low-dose flecainide in CPVT therapy [81–85]. The Class Ic Na_v_1.5 blocker flecainide (IC_50_ approx. 2–7 µM) causes a pro-arrhythmic slowing of AP conduction under conditions of Na_v_1.5 haploinsufficiency including the BrS (review: [86]). However, more recent reports suggesting its partial reclassification as a cardiotropic drug [87–89] were prompted by its additional RyR2 inhibitory actions (IC_50_ approximately 5–11 µM) [85,90,91]. Flecainide (1 µM) exerted respective pro- and paradoxically anti-arrhythmic actions in WT and homozygotic *RyR2-P2328S* atrial preparations. In WT, flecainide (1 µM) reduced loose-patch clamp *I*_Na_ and slowed multi-electrode array recordings of CV while sparing refractory periods (figure 5*d*i,*e*i). In *RyR2-P2328S*, it contrastingly restored *I*_Na_ to WT values, similarly sparing refractory periods (figure 5*d*ii,*e*ii). These effects were replicated by dantrolene challenge (10 µM; figure 5*f*). Higher flecainide concentrations (5 µM) reduced *I*_Na_ in both WT and *RyR2-P2328S* [76]. These findings suggest that the low-dose flecainide rescued *RyR2-P2328S* arrhythmic phenotypes by inhibiting RyR2-mediated Ca^2+^ elevations and their pro-arrhythmic inhibition of Na_v_1.5. These findings may underly its translation into anti-arrhythmic clinical monotherapy for CPVT at low doses [81–85] (however, see also [88,92–95]).

## 9. Possible feedback effects on Na_v_1.5 function in chronic metabolic cardiac pathology

**Figure 6.**
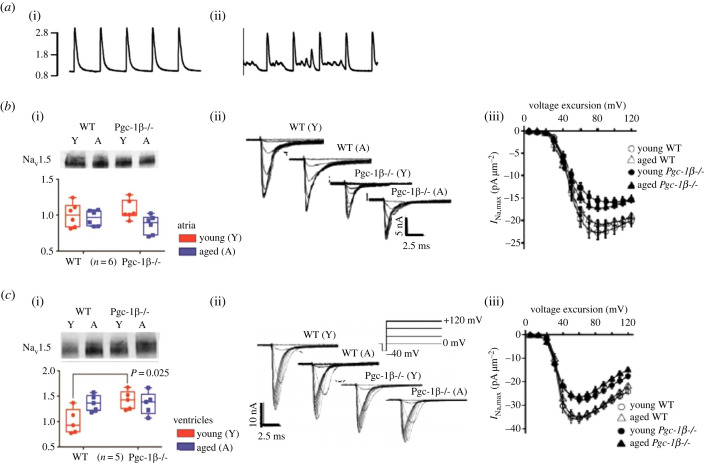
Altered Ca^2+^ homeostasis and *I*_Na_ in peroxisome proliferator-activated receptor-γ coactivator-1 (PGC-1) transcriptional coactivator deficient (Pgc-1β^−/−^) murine cardiomyocytes. (*a*) Line scans averaged from fluorescence confocal microscopy of regularly paced (0.5 Hz), fluo-4 loaded, WT (i) and *Pgc-1β^−/−^* ventricular myocytes (ii). (*b,c*) Results from (i) Western blot assessments of Na_v_1.5 expression, (ii) ion current measurements and (iii) activation current–voltage relationships in young (Y) and aged (A) WT and *Pgc-1β*^−/−^ atria (*b*) and ventricles (*c*). ((*a*) from figure 3A*–*C of Gurung *et al.* [103]; (*b*)(i) from figure 1(A,B) and (*c*)(i) from figure 3(A,B) of Edling *et al.* [104]); (*b*)(ii) from figure 1(E–H) and (*b*)(iii) from figure 1J of Valli *et al.* [105]; (*c*)(ii) from figure 2(e–h) and (*c*)(iii) from figure 2(j) of Ahmad *et al.* [106], by permission.)

Ageing and the age-related conditions obesity, diabetes mellitus and cardiac failure, all associated with perturbed cellular energetics, can manifest in cardiac electrophysiological changes and pro-arrhythmic phenotypes [96,97]. The peroxisome proliferator-activated receptor-γ coactivator-1 (PGC-1) transcriptional coactivators PGC-1α and PGC-1β—highly expressed in oxidative, including cardiac, tissues—are downregulated in these conditions [98]. PGC-1s regulate mitochondrial mass, function and cellular metabolism and upregulate nuclear and mitochondrial gene expression underlying fatty acid β-oxidation, the tricarboxylic acid cycle and electron transport [99]. In addition to increased reactive oxygen species production [100,101] and ATP/ADP depletion [102], recent reports implicate altered Ca^2+^ homeostasis in such pathology. Spontaneous Ca^2+^ leaks similarly occur in atrial fibrillation, cardiac failure and hypertrophic cardiomyopathies. These effects can thus be examined in genetic platforms with modified PGC-1 expression. Isolated *Pgc-1β*^−/−^ murine cardiomyocytes showed altered ion channel expression and function, abnormal Ca^2+^ homeostasis and delayed after-depolarization phenomena, modelling their perturbed electrophysiological function (figure 6*a*i,ii) [103].

*Pgc-1β*^−/−^ hearts showed reduced Na_v_1.5 electrophysiological activity. Western blots of atrial and ventricular tissue lysates from young and old, and WT and *Pgc-1β*^−/−^ mice showed similar Na_v_1.5 expression levels except for actually greater Na_v_1.5 expression in young *Pgc-1β*^−/−^ than WT ventricles (figure 6*b*i,*c*i) [104]. Yet, loose-patch clamped myocytes in superfused *Pgc-1β*^−/−^ atria and ventricles from both young and aged mice showed *I*_Na_ reduced compared to WT (figure 6*b*ii,*c*ii) [105,106]. The corresponding differences in current–voltage relationships related to genotype rather than age (figure 6*b*iii,*c*iii). These findings went with reduced atrial and ventricular (d*V*/d*t*)_max_ and CVs, and increased arrhythmic tendency in Langendorff-perfused hearts on sharp electrode recording [107,108].

## 10. Feedback effects on cardiac Na_v_1.5 function following action potential prolongation

Further extensions of hypotheses invoking Ca^2+^-mediated feedback actions on Na_v_1.4/Na_v_1.5 arise from the reciprocal interactions between surface membrane ion channel activity and Ca^2+^ homeostasis [109]. In addition to variations in upstroke characteristics that result from *I*_Na_ actions, cardiac muscle APs can vary in their recovery durations (APDs) reflecting modifications in their *I*_K_ or *I*_Ca_. One extended Ca^2+^-mediated *I*_Na_ feedback hypothesis might predict that an extended AP depolarization phase associated with an increased APD could enhance RyR2-mediated Ca^2+^ release. This could result in elevated or prolonged [Ca^2+^]_i_ transients that in turn might exert feedback effects on Na_v_1.5 [110].

Recent studies prompted by potential cardiac arrhythmic concerns arising from Covid-19 therapy had investigated effects of HCQ alone and combined with azithromycin (HCQ+AZM). Of these, *in vitro* electrophysiological cultured cell models [111,112] and modelling studies bearing on these drug actions on individual surface membrane ion channels [112,113] predicted potentially pro-arrhythmic behaviour. Experiments integrating biophysical studies of ion channel properties with studies of AP generation and conduction, and of Ca^2+^ homeostatic processes in intact hearts together additionally suggested Ca^2+^-mediated feedback actions on AP generation and conduction [109].

**Figure 7.**
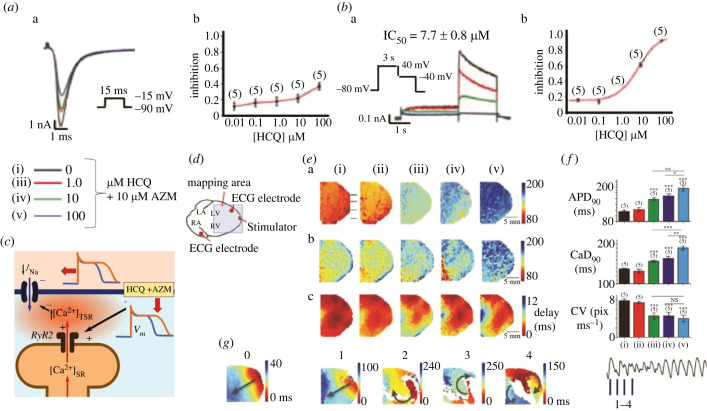
Effects on human (h) Na_v_1.5 function following increases in APD secondary to HCQ and AZM challenge. (*a,b*) Effects of 0, 1, 10 and 100 µM HCQ with 10 µM AZM on hNa_v_1.5 (*I*_Na_, (*a*)) and hERG (*I*_Kr,_ (*b*)), showing (a) currents and (b) consequent concentration-response plots constructed from maximum magnitudes of each current in HEK293 cells. (*c*) Scheme relating these findings to (*d*) schematized mapping experiments in isolated perfused hearts with indicated optical mapping and ECG monitoring configuration. (*e*) Maps of (a) APDs at 90% repolarization (APD_90_) measured using RH237, (b) CaD durations at 90% recovery (CaD_90_) measured by Rhod-2 AM and (c) AP initiation and conduction measured using RH237. (*f*) The resulting (a) APD_90_, (b) CaD_90_ and (c) CV averaged over the field of view. Comparisons in (*e,f*), made (i) before and following challenge by (ii) 1 µM and (iii) 10 µM HCQ, and 10 µM HCQ combined with either (iv) 1 or (v) 10 µM AZM. (*g*) Voltage RH237 optical mapping of re-entry and ventricular arrhythmic tendency in isolated perfused hearts paced at a 170 ms CL, before (control numbered ‘0’) and following addition of 10 µM HCQ with added 10 µM AZM. Each map corresponds to the timepoints numbered 1–4 shown on the monitored ECG. NS not significant, *p < 0.05, **p < 0.01, ***p < 0.001. ((*a*,*b*) from figure 3(A,D)(i) and (ii); (*d*) from figure 6(A)(i); (*e*)(a–c) and (*f*) from figure 6(B)(i) and (iii), (C)(i) and (iii), and (A)(i) and (iv); (*g*) from figure 7(B,C) of Wang *et al.* [109] by permission).

Here, therapeutic HCQ, and particularly (HCQ+AZM), levels inhibited human-expressed *I*_Kr_ and *I*_K1_ but not *I*_to_ and *I*_Ks_. HCQ though not (HCQ+AZM) affected *I*_Na_ and *I*_CaL_ and then only at higher IC_50_s, in patch-clamped HEK293 cells (figure 7*a,b*). Rh237 mapping of membrane potential changes in isolated perfused guinea pig hearts (i) before and following challenge by (ii) 1 µM and (iii) 10 µM HCQ, and 10 µM HCQ combined with either (iv) 1 or (v) 10 µM AZM (figure 7*c,d*) showed that µM-HCQ expectedly increased APD_90_ (figure 7*e*a,*f*a) and APD (figure 7*e*b,*f*b). However, it also reversibly reduced left atrial and ventricular CVs. It additionally increased conduction heterogeneities on multi-electrode array mapping (figure 7*e*c,*f*c). ECGs were bradycardic with increased PR and QRS durations. O'Hara-Rudy modelling reproduced the APD but not the CV alterations from the *in vitro I*_Kr_, *I*_K1_ and *I*_Na_ effects. However, Rhod-2 mapping demonstrated increased durations and dispersions of the intracellular [Ca^2+^] transients. This offers a potential mechanism for the observed downregulation of *I*_Na_ and CV invoking a Ca^2+^-mediated feedback on Na_v_1.5 function. Furthermore, (HCQ+AZM) accentuated all these effects of HCQ. It further disrupted AP propagation and induced alternans and torsades-like episodes during forced pacing (figure 7*g*) [109].

## 11. A broader group of Ca^2+^-mediated feedback effects on membrane excitability?

The present findings suggest Ca^2+^-mediated effects on Na_v_1.4 and Na_v_1.5 that might exert an *in vivo* feedback modulation of excitation–contraction coupling in intact native skeletal muscle fibres and cardiac myocytes. Such actions could form a subclass of a range of feedback mechanisms in which local-elevated microdomain [Ca^2+^] resulting from store Ca^2+^ release might modify surface membrane channel activity and reduce cell excitability [114,115]. The latter in turn may constitute a subcategory of a broader group of Ca^2+^-mediated actions including Ca^2+^-enhanced slow RyR inactivation [116]. Thus, opening Ca^2+^-activated K^+^ channels, whether in skeletal, cardiac or smooth muscle, would hyperpolarize and similarly reduce excitability in their containing membranes. ‘Big K^+^’ (BK) large conductance, Ca^2+^ and voltage-activated, K^+^ channels have intracellular C-termini containing a gating ring comprising two RCK1 and RCK2 domains. These regulate K^+^ conductance through two distinct high-affinity Ca^2+^-binding sites in each domain [117,118]. Their opening requires both membrane depolarization and increased (μM) local [Ca^2+^] [119]. They may decrease membrane excitability in exercising skeletal muscle [66] and influence sinoatrial node firing rate and cardiac pacing *in vivo* [120,121]. Genetic modifications in their encoding *KCNMA1* lead to clinical brain and muscle dysfunction [122,123].

By contrast, small conductance (SK) K^+^ channel, SK1, SK2 and SK3 [124,125], subtypes include a regulatory C-terminal CaM-binding domain. They respond to submicromolar [Ca^2+^]_i_ increases that could arise from RyR-induced Ca^2+^ release [126] but are not sensitive to voltage change [127,128]. Normal adult human and murine, atria and ventricles express SK2 [129]. Its exact physiological cardiac role remains uncertain. It has been suggested to contribute to repolarization in normal healthy atrial [130] though not ventricular cardiomyocytes [131]. Nevertheless it may participate in atrial pathological situations including atrial fibrillation [132]. Furthermore its expression level increases with pro-arrhythmic and hypertrophic changes [133,134] observed in both experimental and clinical ventricular failure [134,135]. Studies combining results from *in vitro* adult and neonatal cardiomyocyte preparations and intact perfused hearts demonstrated increased SK2 expression and pharmacologically detectable effects upon AP recoveries paralleling ventricular hypertrophic changes induced by angiotensin II and p21-activated kinase type 1 downregulation [136]. These proved attributable to direct effects of CaMKII and CREB-mediated signalling on the KCNN2 gene promoter. This [Ca^2+^]_i_-mediated SK regulation could then modify membrane potential or excitability and consequent arrhythmogenicity.

Finally, [Ca^2+^]-sensitivity properties also occur in some anion channels. Thus, Ca^2+^-activated Cl^−^ TMEM16A channels [137], additional to established roles in smooth muscle [138], may also function in cardiac [139] and skeletal muscle [140].

## Data Availability

This article has no additional data.
